# Carcinosarcome de la voie biliaire principale: à propos d’un cas

**DOI:** 10.11604/pamj.2024.48.190.43645

**Published:** 2024-08-30

**Authors:** Khalid Rabbani, Tariq Ahbala, Mohamed Boulatar, Abdellatif Nebgui, Abdelouahed Louzi

**Affiliations:** 1 Service Chirurgie Générale Hôpital Arrazi, Centre Hospitalo-Universitaire Mohammed VI, Marrakech, Maroc,; 2 Faculté de Médecine et de Pharmacie Marrakech, Université Cadi Ayad, Marrakech, Maroc

**Keywords:** Ictère cholestatique, voies biliaires, carcinosarcome, cas clinique, cholestatic jaundice, common bile duct, carcinosarcoma, case report

## Abstract

Les carcinosarcomes des voies biliaires sont des tumeurs très rares. Leurs diagnostics et leurs prises en charge demeurent difficile. Nous rapportant le cas d'un patient chez qui le diagnostic a été suspecté devant un ictère cholestatique associé à une altération de l'état général. Biologiquement, la bilirubine, la phosphatase alcaline (PAL) et les gamma-glutamyl transférase (GGT) étaient élevés. L'imagerie a montré une tumeur de la tête du pancréas. L'exploration chirurgical, la biopsie tumorale et l'étude anatomo-immunohistochimique ont confirmé l'origine biliaire. Le but de notre cas est de montrer la difficulté du diagnostic et de la prise en charge ainsi que l'importance de l'étude anatomopathologique et immunohistochimique pour la confirmation du diagnostic.

## Abstract (en)

Carcinosarcomas of the bile ducts are very rare tumors. Their diagnosis and management remain difficult. We here report the case of a patient in whom the diagnosis was suspected due to cholestatic jaundice associated with a deterioration in general condition. Biologically, bilirubin, alkaline phosphatase (ALP) and gamma-glutamyl transferase (GGT) levels were elevated. Imaging showed a tumor in the head of the pancreas. Surgical exploration, tumor biopsy and anatomo-immunohistochemical study confirmed the biliary origin. The purpose of our case report is to highlight the difficulties in diagnosis and management as well as the importance of anatomopathological and immunohistochemical studies in confirming the diagnosis.

## Introduction

Le carcinosarcome est une tumeur maligne rare, associant un contingent épithélial carcinomateux et un contingent mésenchymateux malin pouvant renfermer un tissu hétérologue [1]. Il survient au niveau de plusieurs organes tels que l'utérus, la vessie, le poumon, l'œsophage [1,2]. La localisation au niveau de l'arbre biliaire est rare [2]. Elle a été décrite pour la première fois en 1907 par Landsteiner [1]. Nous rapportant le cas d'un carcinosarcome de la voie biliaire principale.

## Patient et observation

**Informations du patient:** il s'agit d'un patient âgé de 56 ans, sans antécédents pathologiques particuliers. Le patient a été admis au service de chirurgie générale du Centre Hospitalier Universitaire Mohammed VI de Marrakech, pour la prise en charge d'un ictère d'allure cholestatique associé à une altération de l'état général.

**Résultats cliniques:** l'examen clinique à objectivé un patient conscient, stable sur le plan hémodynamique et respiratoire, OMS: 1 et IMC: 19.2 ictère cutanéo-muqueux généralisé avec lésion de grattage et légère sensibilité épigastrique, le toucher rectale était sans anomalie.

**Chronologie:** le débute de la symptomatologie remonte à 1 mois par l'installation progressive d'un ictère d'allure cholestatique fait d'urines foncés et selles décolorés associé à un prurit sans autres signes digestifs ou extra-digestifs. Le tout évoluant dans un contexte d'apyrexie et altération de l'état général fait d'anorexie et d'amaigrissement non chiffré.

**Figure 1:**
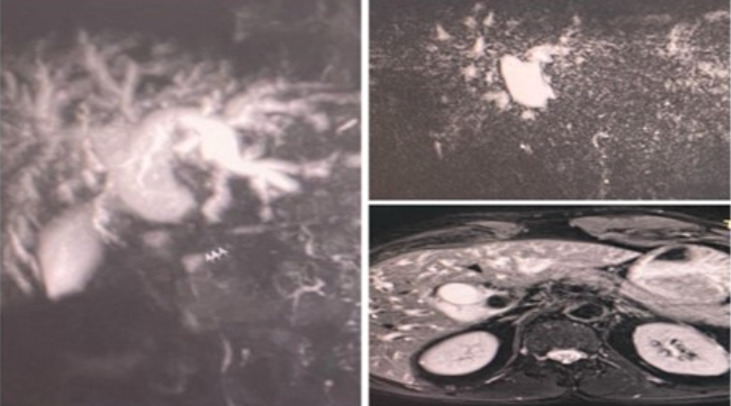
différentes coupes de l'imagerie par résonnance magnétiques biliaire montrant la tumeur

**Figure 2:**
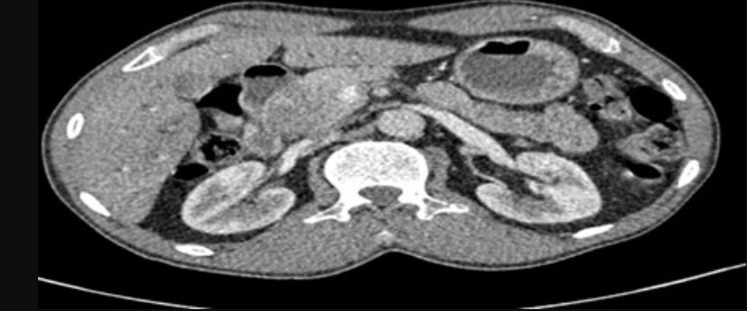
aspect scanographique de la tumeur pancréatique

**Démarche diagnostique:** l'examen biologique mettait en évidence une bilirubine totale à 262 à prédominance direct 227, un syndrome de cholestase (PAL/GGT: 421/98) et une CA19-9 à 5396 (200 fois la normale). La Bili-IRM objectivait un processus lésionnel de la tête du pancréas mesurant 29.5x24.3mm, discrète dilatation du conduit pancréatique principal mesurant 3.2mm et importante dilatation des voies biliaires intra-hépatiques et de la voie biliaire principale mesurant 22mm, présence des ganglions infracentimétriques en inter-aortico-cave et absence de lésion hépatique (Figure 1). La tomodensitométrie (TDM) abdominal montrait un processus de la tête du pancréas mesurant 38x32x38, englobe la voie biliaire principale mesurant 25mm avec une dilatation majeure d'amont et des voies biliaires intra-hépatiques. Ce processus englobe en dedans la veine mésentérique supérieure et la confluence portale sur une circonférence de 95° qui reste perméable et infiltre partiellement la lame rétro portale et respect du troncs cœliaques et de l'artère mésentérique supérieur; en arrière arrive au contact de la veine cave inférieure, du pédicule droit et la veine rénale gauche avec perte de liseré graisseux de séparation et qui restent perméables; en haut arrive au contact de la bifurcation de l'artère hépatique commune et englobe l'artère duodéno-pancréatique; présence de quelques ganglions para gastriques droits, latéro aortiques et iliaques primitifs infracentimétrique; le foie était normal et présence des lésions ostéolytiques type Ib de Lodwick au niveau du corps vertébrale de L4 et du col fémoral gauche et Ia au niveau de l'aile iliaque gauche avec quelques remaniements dégénératifs. La TDM thoracique n'objectivait aucune lésion secondaire (Figure 2).

**Figure 3:**
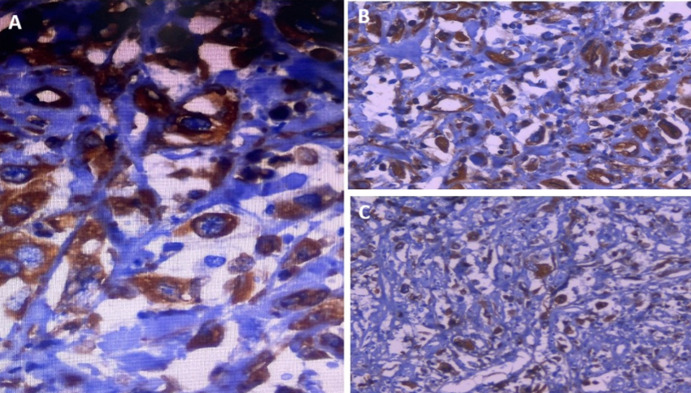
anatomopathologie et immunohistochimie: A) tumeur à cellules pléiomorphes indépendantes au niveau de la voie biliaire principale (VBP); B) expression cytoplasmique intense des cellules tumorales pléiomorphes de l'anticorps anti-vimentine; C) expression cytoplasmique intense des cellules tumorales pléiomorphes de l'anticorps anti-cytokératine 19

**Intervention thérapeutique:** une duodénopancréatectomie céphalique était envisagé mais à l'examen macroscopique objectivait une tumeur de la voie biliaire principale au niveau du cholédoque en aval de l'insertion de canal cystique de 6cm de grand axe infiltrant le pédicule hépatique et le tronc cœliaque et arrivant au contact de la tête du pancréas sans l'envahir; une voie biliaires principale dilatée à 3cm; pas d'ascite, pas de carcinose, pas de métastase hépatique. Le geste a consisté à une biopsie de la tumeur et confection d'une dérivation cholédoco-duodénale. L'examen anatomopathologique montrait une prolifération tumoral maligne indifférenciée à cellules pléomorphes nécessitant un complément immunohistochimique et une cholécystite chronique diverticulaire avec foyers d'hyperplasie adénomyomateuse de la vésicule biliaire, sans signe histologique de malignité. L'immunohistochimie objectivait un carcinome peu différencié et infiltrant de type sarcomatoïde (carcinosarcome) (Figure 3).

**Suivi et résultats des interventions thérapeutiques:** les suites post-opératoires ont été marqué par une régression clinique de l'ictère et du prurit avec diminution des valeurs biologiques de la bilirubine, GGT et PAL. Le patient était adressé au service d'oncologie pour éventuelle chimiothérapie, les suites étaient marquées par le décès du patient après 2 séances de chimiothérapie.

**Perspectives du patient:** le patient était satisfait de la bonne évolution clinico-biologique et non du traitement palliatif.

**Consentement éclairé:** le patient a donné son consentement librement et de façon éclairée, afin de permettre la réalisation et la publication de ce manuscrit.

## Discussion

Les carcinosarcomes sont caractérisés par la présence de composants épithéliaux et mésenchymateux au sein de la même tumeur. Le site le plus fréquent de carcinosarcome des voies biliaires est la vésicule biliaire, avec moins de 100 cas signalés dans la littérature; comparativement, il y a seulement 7 cas de carcinosarcome des voies biliaires signalés dans la littérature anglaise à ce jour [1]; le cas de notre cas rapporté.

La pathogenèse de ces tumeurs rares est incertaine mais plusieurs hypothèses existent. On a théorisé que le carcinosarcome provient de cellules souches stromales totipotentes capables de différenciation divergente. Une autre postulation est la théorie des tumeurs de collision qui suggère la prolifération maligne distincte et simultanée des composantes épithéliales et mésenchymateuses dans le même tissu. Il a également été suggéré qu'un carcinome peut se transformer en sarcome par transformation métaplastique [1-3]. Une caractéristique distinctive d'un véritable carcinosarcome est la nature biphasique de la tumeur avec un manque de transition entre les deux composantes épithéliales et sarcomateuses par opposition à un carcinome mal différencié avec motif de cellules fuseau [1,2]. L'élément sarcomateux est généralement constitué de cellules fuseaux indifférenciées et d'une variété de composants hétérogènes tels que, chondro-, ostéo-, leiomyo-, rhabdomyosarcome. L'élément épithélial est habituellement constitué d'un adénocarcinome et, à l'occasion, de composants tels que les carcinomes épidermoïdes, à petites cellules et indifférenciés [4,5]. L'analyse génétique a été utilisée pour évaluer la pathogenèse de ces tumeurs. Ils ont également été subdivisés en deux sous-groupes: l'un avec prédominance sarcomateux ou à prédominance carcinomateuse [6].

Les caractéristiques démographiques du carcinosarcome biliaire sont semblables à celles de l'adénocarcinome de la vésicule biliaire, la majorité survenant chez les femmes âgées et fortement associée à la lithiase biliaire [7]; le sexe de notre malade est masculin et la vésicule biliaire était libre. Le pronostic est sombre, largement extrapolé à partir de l'expérience des carcinosarcomes de la vésicule biliaire. La durée médiane de survie rapportée des patients atteints de carcinosarcomes de la vésicule biliaire après la résection chirurgicale n'a duré que 7 mois, avec des taux de survie à 1, 2 et 3 ans de 37,2%, 31,0% et 31,0% respectivement [8]. Dans notre cas la survie était de 3 mois après traitement palliatif.

En général, les carcinosarcomes sont des tumeurs localement agressives avec une propension à métastases systémiques même dans les premiers stades [9]. Dans notre cas la tumeur envahie les organes de voisinage. Il a été suggéré que l'agressivité de la tumeur dépend du sarcomatoïde composant, car ils métastasent aux ganglions lymphatiques et aux organes distants plus facilement. Aussi la composante sarcomatoïde qui forme la majeure partie de l'élément polypoïdal qui conduit à une présentation plus précoce car il obstrue les canaux biliaires, contrairement aux carcinosarcomes de la vésicule biliaire, où l'élément carcinomatoïde forme la composante infiltrative de la tumeur [1,4]. En raison de la rareté du carcinosarcome des voies biliaires, son vrai pronostic et ses caractéristiques cliniques ne sont pas bien établis.

La prise en charge de toutes les tumeurs malignes biliaires, y compris le carcinosarcome, demeure difficile, et cela est particulièrement vrai pour celles qui proviennent de l'arbre biliaire proximal. La stratégie a été largement extrapolée à partir de l'expérience acquise. Dans le traitement du cholangiocarcinome, avec la résection comme base du traitement. Comme pour l'adénocarcinome biliaire le plus courant, rien n'indique clairement que la chimiothérapie ou la radiothérapie apporte un bénéfice sur la survie, que ce soit en traitement adjuvant ou en traitement palliatif [1,9,10]. Cependant, juger par les résultats médiocres, il est clair que la chirurgie seule est inadéquate. Une chimiothérapie adjuvante a été utilisée pour traiter les carcinosarcomes des voies génitales féminines. Mais avec des résultats décevants et le rôle du rayonnement est actuellement incertain [1,9]. Notre patient a reçu un traitement palliatif fait d'une dérivation cholédoco-duodénal puis une chimiothérapie adjuvante.

## Conclusion

Le carcinosarcome du canal biliaire est une tumeur rare, son diagnostic est difficile et seul l'étude anatomopathologique et immunohistochimique qui peuvent la différenciée des cholangiocarcinome. Le traitement optimal est la résection complète si c'est possible. La chimiothérapie et/ou la radiothérapie est indéterminée et le pronostic est sombre.
